# Molecular and serologic diagnostic approaches; the prevalence of herpes simplex in idiopathic men infertile

**Published:** 2014-05

**Authors:** Nasser Amirjannati, Farhad Yaghmaei, Mohammad Mehdi Akhondi, Mahboubeh Nasiri, Hamed Heidari-Vala, Zahra Sehhat

**Affiliations:** 1 *Reproductive Biotechnology Research Center, Avicenna Research Institute, ACECR, Tehran, Iran.*; 2 *Monoclonal Antibody Research Center, Avicenna Research Institute, ACECR, Tehran, Iran.*; 3 *Nanobiotechnology Research Center, Avicenna Research Institute, ACECR, Tehran, Iran.*

**Keywords:** *Herpes virus*, *Infertility*, *IgM*, *Polymerase Chain Reaction*, *Semen*, *Serologic*, *Sexually transmitted diseases (STD)*

## Abstract

**Background:** Human pathogens that can cause infertility may also affect sperm count and quality. Viral infections can be considered as direct and/or indirect cause of male factor infertility.

**Objective:** Our goal was to investigate the prevalence of herpes simplex virus in the semen of infertile men attending the Avicenna Infertility Clinic, and to compare it with the herpes virus serology results.

**Materials and Methods: **This cross sectional study was conducted during 2009-2010. Infertile men participating without any clinical signs of infection with herpes simplex virus, and no obvious cause for their infertility were included. Semen and blood samples were used for Polymerase Chain Reaction (PCR) and serologic testing for these people. Two samples were collected: one ml semen sample to verify the existence of genital herpes simplex virus in infertile men, and blood samples of 217 individuals tested for antibodies to herpes simplex virus. Data were analyzed by SPSS 16.

**Results: **According to the PCR results of semen samples the prevalence of herpes simplex in semen was 12% and serologic test showed 3.2% prevalence within blood. Nine to 10% of IgM negative were PCR positive and only 2-3% of IgM positive were PCR positive. Between herpes serologic studies with positive controls and negative controls by using both tests, there was a significant positive relationship (r=0.718 and p<0.001). The relationship between semen PCR test results and serological survey of herpes patients with a negative control in both Pearson and Spearman tests was positive and significant (r=0.229 and p=0.001). Correlation between the PCR results of semen samples with two positive control subjects and a positive IgM test was statistically confirmed (r=0.235 and p<0.001).

**Conclusion:** We recommend that if there is suspicion to herpes simplex as a microorganism that theoretically could impact semen parameters and cause infertility it is prudent to use PCR technique on semen sample rather than ELISA on serum.

## Introduction

Today, despite advances in the reproductive biotechnology area, infertility has remained until as one of the most important health problems in different communities. It can cause communication problems, social stigma, divorce and other social and psychological consequences (1-3). In Europe, about 15-20% of couples living within reproductive age are affected by infertility. In the case of about one-third of infertile couples, the men and women are equally affected and in 35-50% related to male factor infertility (2, 4-8). Several studies have shown that about 30-75% of cases without specific causes could be associated to male factor (5, 9). Idiopathic cases of infertility, because unknown factors which interfere with the therapeutic models, cannot have successful treatment (2, 10, 11). There are different causes of male infertility, including spermatogenesis disorders, chronic diseases, sexual dysfunction, germ line distress following therapeutic complications such as chemotherapy and radiotherapy and occupational exposure to chemical or physical deleterious matters (12-14).

Studies in this field have proved that human pathogens can cause infertility and may also affect sperm count and quality so that bacterial infections can lead to male factor infertility in 6.6-48% of cases (5). Viral infections also can lead to direct and indirect male factor infertility. Pathogens can induce reproductive disorders through toxic and immunological reactions in the genital ducts. Since viruses can be detected in cell-free semen sample, spermatozoa and white blood cells, it seems that semen can be the putative route of viral transmission (15-17).

In spite of common infections including chlamydiosis and major of sexual transmitted infections can affect fertilizing ability, HIV cause no changes in semen parameters including pH, sperm concentration, morphology, vitality and sperm motility. In contrast, HPV (human papiloma virus) through human-specific genes can interfere with sperm motility. Hepatitis C and B viruses are harbored in semen and male genital ducts to play a role as reservoir for the virus, opportunistically (18-21).

In semen of infertile men with no symptoms of infection, DNA of Herpes-viridae family including herpes simplex virus types 1 and 2 has been detected (15-17, 21). Numerous studies have examined the impact of viral infections such as CMV (cytomegalovirus), HSV, and EBV (Epstein-Barr virus) on mechanisms of oxidative stress on sperm parameters but only HSV has had an active role in the creation of such damage (22). Studies have verified herpes simplex virus I and II in semen samples from 4-50% of infertile men through IgM antibodies and leukocytospermia (23-25).

Many cases with genital herpes have been observed that the symptoms are atypical or their culture is negative. They have no anti-herpes antibody in acute phase. But it should be note that only in recovering period the serum antibody titer of the virus can be seen by serological methods (26). On the other hand genital herpes can lead to considerable morbidity and even some neonatal infectious complications such meningitis (27). Antibodies could be detected in patient’s serum after 4-7 days from infection emergence when is considered as time point for serological tests. Regarding to necessity of precise timing for anti-viral therapy the rapid diagnosis seems to be performed. Therefore, serologic tests cannot be used as an absolute indicator. In addition to the mentioned methods, Western blot analysis also can be used as a highly sensitive and specific test for diagnosis of virus, but due to the high professional level, lack of access, time-consuming and high cost, the application of this method is limited. The PCR technique is a method for detection of small quantities of virus in clinical samples (26, 28, 29).

Different studies have demonstrated that the virus is associated with the occurrence of infertility in men; Aynaud *et al* identified the presence of Herpes Simplex Virus (HSV) in sperm of men with genital infection using PCR (2, 30-32). Wald and colleagues by PCR observed that 47% of samples that are serologically negative have herpes simplex virus DNA (33). Borai *et al* showed that this virus is associated with infertility (34) and in the other study by Foresta *et al* and based on data obtained from in situ hybridization technique HSV was detected in spermatozoa and its association with infertility was clearly defined (35). Also Huttner *et al* study that was performed on transgenic mice, emphasize the relationship between HSV and infertility, more and more (30, 31).

Kapranos and colleagues in a study on 113 semen samples of infertile men, using a nested PCR technique, found significant correlation between the herpes virus prevalence and infertility due to oligo/ astenozoospermia (2). The prevalence of herpes virus in the male reproductive ducts in various studies using different methods has shown a range 3-49.5%. Almost all the studies have emphasized the relationship between herpes infestation and changes in sperm parameters (2, 21, 24). Perhaps herpes virus is considered as one of the suspected reasons for Assisted Reproduction Techniques (ART) failure. Therefore, PCR can reduce the cost of repeated treatments in these patients. 

For those who have undergone infertility treatments, the better the screening test in the early stages, the sooner to find a specific cause for infertility. Todd and colleagues showed that prevalence of detectable genital HIV RNA copies varied from 73% in HSV-2 seronegative women to 94% in women with herpetic lesions (35). However regarding to all what we know so far about significant association proved between HSV and HIV promote clinical researchers to plan strategies predicting individuals with high risk status in develop and transmitting HIV (27). To help for making fit policy in infectious diseases such HSV control we have to achieve a comprehensive epidemiologically vision and dominance. Therefore this study aimed to compare seroprevalence and molecular methods to offer sensible comments in control strategies.

## Materials and methods

This is a descriptive study supported by Reproductive Biotechnology Research Center, Avicenna Research Institute, ACECR, Tehran, Iran about men suffered idiopathic infertility and referred to the Avicenna Infertility Clinic throughout years 2009 to 2010. Also, present study procedures were approved by local bioethics committee of Avicenna Research Institute. The entire of study procedure has been supervised with Avicenna Research Institute Bioethics Committee. Considering the d=0.04 and p=10% and Z=1.96 in the formula for sample size in the descriptive study, 217 samples were considered. 

Men participating had no clinical signs of HSV infection and no obvious causes for their infertility were found. All of them were infertile due to un-known causes. Individuals with any underlying diseases such diabetes which can likely interfere with fertility potential were excluded. They then were informed about study and were ensured that non-interference present between this study and their treatment procedure. Also confidentiality of participant’s information was guaranteed. Eventually after informed consent forms were signed, volunteers were included in study. Samples of blood and semen which had been collected during routine diagnostic procedures were used. Then participants were asked to fill the questionnaire containing basic demographic information, history of marriage, sex and risky behavior alongside interviews scheduled for urology ward consultations. 

Two types of samples were collected: 217 semen samples (1ml) to verify the genital herpes simplex virus and 217 blood serum samples to test the antibodies to herpes simplex virus. Blood samples after coagulation, treated for one hour in the 37^o^C and then centrifuged for 10 min in 2200 g and serum obtained to measure the titers of HSV (I and II) antibodies. In order to test the HSV antibodies on serum samples, ELISA was performed by the EUROIMMUN kit (EI 2531-9601-1 M). After preparing the plates for the ELISA test, the range of samples was set to wavelength of 450nm. The data obtained from each sample spectra using following formula leaded to final results.

Ratio= OD Sample/ OD Calibrator

Negative: Ratio<0.8

Borderline: 0.8≤Ratio<1.1

Positive: Ratio≥1.1

In the case of PCR test of semen, firstly sample DNA was extracted using DNA purification from fluid Semen kit (QIAGEN; Cat No: 57704) according what company had advised. In continue, PCR test was carried out with Herpes Simplex Virus Detection Kit (CINNAGEN; Cat No: PR8240C) as like as kit protocol.

**Statistical Analysis**

The data collected were analyzed with SPSS software version 16. The Pearson and Spearman tests for correlation between variables with a significance level of 0.01 were used. The data were expressed as Mean±SD.

## Results

Two hundred seventeen infertile men referred to the Avicenna Infertility Clinic, had been selected to participate in this study. The average age of participants was 33.84±5.619. Their age range was 19-49 years. 4.1% was uneducated, 21.7% had secondary school education and 74.2% had college degrees. Condition of employment was as follows: 38.7% of participants in the study were employee, 22% were self-employed, 19.4% workers and 0.5% had no job. 

The rest were in other occupational groups. 89.9% of people surveyed were living in urban and the remaining in rural areas. Of those who were studied, 98.6% were married and the rest were unmarried. Maximum marital duration of participants were 26 years and average duration of marriage, was 7.56±5.318 year and 7.4% had remarried. Classify people based on a frequency of remarriage 5% for once and 1.48% has remarried twice. 1.8% of women had experienced a previous marriage. First sexual experience age range was 10-28 and the average was 15.72±2.605 years. 

The recent history of sex in 96.3% of the sample was found.42% of subjects had anal/oral sex. 6% of cases had a history of blood transfusion. Those who had STD were 2.8% and in all of them herpes IgM test was negative. Serology results had shown 3.7% IgM positive, 3.2% borderline IgM titer and 93.1% IgM negative. Results of serum IgM result based on STD, blood transfusion and anal/oral sex history are summarized in Table I. In addition, PCR results of seminal fluid considering STD, blood transfusion and anal/oral sex history are shown in Table II.

According to the PCR results of semen samples the prevalence of herpes simplex in semen and blood samples was 12% and 3.2% respectively. Results obtained from PCR and ELISA in semen and serum of men with idiopathic infertility is shown in Table III. Nine to 10% of IgM negative were PCR positive and only 2-3% of IgM-positive were PCR positive. Pearson and Spearman correlation test with a significance level of 0.01 was used. Between herpes serologic studies with positive controls and negative controls by using both tests, there was a significant positive relationship (r=0.718 and p<0.001). The relationship between semen PCR test results and serological survey of herpes patients with a negative control in both Pearson and Spearman tests was positive and significant (r=0.229 and p=0.001). 

Spearman's correlation test between the PCR results of semen samples with two positive control subjects and a positive IgM test was statistically confirmed (r=0.235 and p<0.001). Spearman test showed a statistically positive and significant relationship between the age and duration of the marriage with HSV positiveness (r=0.633 and p<0.001). Using both statistical tests, none of the variables were associated with age of first sex. There was no significant statistical association between PCR results, serological tests and age, marital duration, age of first sexual experience.

**Table I:** Results of serological tests in infertile men having a STD, blood transfusion and anal/oral sex history

**IgM**	**History of STD infection**		**History of blood transfusion**		**History of anal /oral sex**		**Sum**
	**Yes**	**No**	**Yes**	**No**	**Yes**	**No**	
Negative (<0.8)	6 (2.8%)	196 (90.3%)	9 (4%)	193 (88.9)	84 (39.1%)	117 (54%)	202 (93.1%)
Borderline (0.8-1.1)	0	7 (3.2%)	3 (1%)	4 (1.8%)	3 (1.4%)	4 (1.8%)	7 (3.2%)
Positive (>1.1)	0	8 (3.7%)	3 (1%)	5 (2.3%)	3 (1.4%)	5 (2.3%)	8 (3.7%)
Sum	6 (2.8%)	211 (97.2%)	15 (6%)	202 (94%)	91 (41.9%)	126 (58.1%)	217 (100%)

Date are presented as n (%).

**Table II:** PCR results in semen of infertile men with STD, blood transfusion and anal/oral sex history

**Semen PCR**	**History of STD infection**		**History of blood transfusion**		**History of anal /oral sex**		**Sum**
	**Yes**	**No**	**Yes**	**No**	**Yes**	**No**	
Negative	5 (2.3%)	186 (85.7%)	4 (1.8%)	187 (86%)	83 (38.2%)	108 (49.8%)	191 (88%)
Positive	1 (0.5%)	25 (11.5%)	11 (4.2%)	15 (7.5%)	8 (3.7%)	18 (8.3%)	26 (12%)
Sum	6 (2.8%)	211 (97.2%)	15 (6%)	202 (94%)	91 (41.9%)	126 (58.1%)	217 (100%)

Date are presented as n (%).

**Table III:** PCR results in semen of infertile men with IgM positive serologic result and negative control

**IgM titers**	**Positive** **control**		**Negative control**		**Sum**
**Semen PCR**	**Positive**	**Negative**	**Positive**	**Negative**	
Negative	9 (4.1%)	182 (83.9%)	4 (2%)	187 (86%)	191
Positive	6 (2.8%)	20 (9.2%)	4 (2%)	22 (10%)	26
Sum	15 (6.9%)	202 (93.1%)	8 (4%)	209 (96%)	217

Date are presented as n (%).

**Table IV:** Results of Pearson test, correlation between variables and IgM titer negative control, and age of first sexual experience

**Pearson** **test**	**IgM** **titers** **with negative control**		**Age of** **first** **sex**	
	**p-value**	**r**	**p-value**	**r**
Age	0.735	0.023	0.324	-0.067
Duration of marriage	0.947	-0.005	0.305	-0.070
IgM titer with positive control	0.000*	0.718	0.368	-0.061
PCR	0.001*	0.229	0.980	0.002
IgM titers with negative control	-	-	0.605	-0.035
Age of first sexual experience	0.605	-0.035	-	-

* Level of 0.01 was considered significant. (Pearson and Spearman test)

**Table V:** Results of Spearman test, correlation with age, duration of marriage, with IgM titers of positive control

**Pearson** **test**	**Age**		**Duration of marriage**		**IgM** **titer** **with positive** **control**		**PCR**		**IgM** **titers with** **negative control**		**Age at first** **sex**	
	**p-value**	**R**	**p-value**	**R**	**p-value**	**R**	**p-value**	**R**	**p-value**	**R**	**p-value**	**R**
Age	-	-	0.000*	0.633	0.069	0.315	-0.050	0.463	0.017	0.808	-0.046	0.500
Duration of marriage	0.000*	0.633	-	-	0.420	-0.055	0.937	0.005	0.958	-0.004	0.392	-0.059
IgM titer with positive control	-0.069	0.315	0.420	-0.055	-	-	0.000*	0.235	0.000*	0.718	0.286	-0.073
PCR	-0.050	0.463	0.937	0.005	0.000*	0.235	-	-	0.001*	0.229	0.398	0.058
IgM titers with negative control	0.017	0.808	0.958	-0.004	0.000*	0.718	0.001*	0.229	-	-	0.480	-0.048
Age of first sexual experience	-0.046	0.500	0.392	-0.059	0.286	-0.073	0.398	0.058	0.480	-0.048	-	-

* Level of 0.01 was considered significant. (Pearson and Spearman test)

## Discussion

The number of couples applying for ART has increased and some of these people are suffering from idiopathic infertility. Due to the presence of many diseases including sexually transmitted diseases as a cause of infertility that has been confirmed by various researchers around the world, the question is that what role pathogens such as herpes simplex play in infertility. The prevalence of HSV-2 and HSV-1 infections varies markedly by country, region and ethnics (27). Given the low number of studies to verify relationship between infertility and these infections, this survey was done to study the prevalence of herpes simplex in the semen of men with idiopathic infertility, to be determined by PCR, then they compared with prevalence of herpes serology tests. Prevalence of herpes simplex in semen samples was 12% and prevalence of herpes simplex in blood samples using serological survey was approximately 3.2%.

Neofytou *et al* studied the prevalence of different types of herpes simplex virus in semen of infertile men and the impact of different types of the virus on sperm parameters, reported 2.5% prevalence of type 1 virus in 172 cases which in comparison to our study is about 10 percent less, this variation comes probably from smaller sample size (4). In Bezold and colleagues study within 6 years that was performed on 241 infertile men the prevalence of herpes reported about 18.7% (36). A 22.86% prevalence of herpes simplex virus type I also reported in Salehi-vaziri *et al* study. The reason of this difference could be related to the type of PCR techniques used (24). 

In Kapranous and colleagues study, the prevalence of HSV was approximately 56.6% in 113 samples (2). Wald and colleagues studied 15 men with clinical signs of HSV infection; semen culture and PCR were done and observed that 47% of them have a negative culture but positive PCR results (33). In our study about 9% difference between ELISA and PCR results were observed. It seems that difference in discrepancy was due to this fact that their sampling was done in symptomatic patients. As can be seen in the prevalence concluded in different studies, differences are significant; these discrepancies could be due to variety in Para clinical methods used, sample size, the population studied, the frequency of sampling and sample storage conditions.

In this study, because the limited number and age distribution of studied subjects, age groups have not been classified so the possibility of more exact survey did not exist. That might explain the lack of statistically significant correlation between age and the prevalence of herpes simplex. In contrast, Smith and colleagues in a study conducted in Poland, reported that the HSV type 2 prevalence increases with age, this increase was about three times since age 40 (from 4-12%) (37). It seems that the sample size and wide geographic area of the serological survey was responsible in variations. In the present study, approximately 9-10% of cases were IgM negative and PCR positive and only about 2-3% of IgM and PCR positive synchronically. The relationship between PCR results of semen samples and serological survey of infected patients were positive and significant. 

Wald and colleagues in a study on 15 men with clinical symptoms of herpes infection showed 47% of people despite having a negative culture, had a positive PCR test but it appears to contain a bit exaggerated, because of the results obtained may be related to samples obtained as early as emergence of initial levels of antibodies after infection (33). On the other hand, as previous HSV-1 infection can decreases the infection severity and probably prohibit HSV-2 infection it appears that there is immunological cross reaction molecular method could be considered preferentially (38). 

In this study, there is a positive and statistically significant relationship between age and duration of marriage with HSV prevalence. None of variables were associated with age of first sexual experience. Despite the large number of studies examined the causes of infertility, affective role of microorganisms in the male infertility has not been clearly recognized so far (36). 

## Conclusion

In conclusion, whenever there is suspicion to herpes simplex as a microorganism that theoretically could impact semen parameters and fertility potential, it is prudent to use PCR technique on semen sample rather than ELISA on serum. In addition in the case of variation between serologic result and clinical demonstration signifying sexually transmitted diseases as like as Herpes simplex, a molecular approach such PCR would be more reliable base for therapeutics strategy design. We also recommend future research plan to survey more accurate comparison of sperm parameters in infertile men with and without HSV. We can conclude that more study for the relationship between education, awareness of couples about STDs and their impact on reproductive health seems to be necessary. Also, the correlation between HSV and HIV predisposing directs clinicians to minimize HSV infection (especially type 2) prevalence trend through prohibitory strategies.
